# Effect of acupuncture at Zusanli (ST36) point on antral contraction function under ultrasound guidance: study protocol of a randomized controlled trial

**DOI:** 10.1186/s13063-021-05704-9

**Published:** 2021-11-15

**Authors:** Yingqi Chen, Yu Bian, Shanshan Li, Yuanyuan Zhao, Jiaying Li, Yuanyi Zheng, Jie Chen, Shifen Xu, Yiqun Mi

**Affiliations:** 1 Shanghai Municipal Hospital of Traditional Chinese Medicine, Shanghai University of Traditional Chinese Medicine, Shanghai, 200071 China; 2 Department of Acupuncture, Massage and Traditional Medical Traumatology and Orthopedic, Shanghai Jiao Tong University Affiliated Sixth People’s Hospital, Shanghai, 200233 China; 3 Department of Ultrasound in Medicine, Shanghai Jiao Tong University Affiliated Sixth People’s Hospital, Shanghai Institute of Ultrasound in Medicine, Shanghai, 200233 China

**Keywords:** Acupuncture, Zusanli, Antral Contraction Function, Ultrasound

## Abstract

**Background:**

Although the relationship between *deqi* sensations and curative effect has always been controversial, *deqi* sensations has been regarded as the key indicator of clinical efficacy of acupuncture therapy. There is little evidence for standardization or visualization of the mechanism of acupuncture’s therapeutic effect. This trial aims to evaluate the effect of needling at Zusanli (ST36) on antral contraction function as visualized by ultrasound.

**Methods:**

This is a two-arm, single-blind, randomized, controlled trial. A total of 116 acupuncture-naïve healthy subjects will be randomly allocated to the acupuncture group or sham acupuncture group in a 1:1 ratio. Participants in the acupuncture group will receive manual acupuncture at Zusanli (ST36) with the needling depth at crural interosseous membrane. Those in the sham acupuncture group will be given penetrating needling depth at the superficial fascia layer. The primary outcome will be the changes in antral contraction frequency (ACF) before and after acupuncture. The secondary outcomes will be the changes in the thermal infrared spectrum of gastric area skin, the antral contraction amplitude (ACA), the antral movement index (AMI), and the scores on the Chinese version of Massachusetts General Hospital Acupuncture Sensation Scale (C-MASS). The adverse events will be evaluated and recorded in detail.

**Discussion:**

This study may provide visual and objective evidence regarding the safety and efficacy of manual acupuncture at Zusanli (ST36). In addition, the results of this study will help to identify the role of Zusanli (ST36)in the inducing *deqi*.

**Trial registration:**

Chinese Clinical Trial Registry ChiCTR2000040686. Registered on 8 December 2020

**Supplementary Information:**

The online version contains supplementary material available at 10.1186/s13063-021-05704-9.

## Administrative information

Note: the numbers in curly brackets in this protocol refer to SPIRIT checklist item numbers. The order of the items has been modified to group similar items (see http://www.equator-network.org/reporting-guidelines/spirit-2013-statement-defining-standard-protocol-items-for-clinical-trials/).

**Table:** 

Title {1}	Effect of Acupuncture at Zusanli Point on Antral Contraction Function under Ultrasound Guidance: Study Protocol of a Randomized Controlled Trial
Trial registration {2a and 2b}	ChiCTR2000040686. Registered on 8th December 2020
Protocol version {3}	version 1.1(22 September 2020)
Funding {4}	Shanghai Hospital Development Center (Grant SHDC2020CR3015A) and the National Natural Science Foundation of China (Grant NO. 81871360).
Author details{5a}	^1^Shanghai Municipal Hospital of Traditional Chinese Medicine, Shanghai University of Traditional Chinese Medicine, Shanghai 200071, China ^2^Department of Acupuncture, Massage and Traditional Medical Traumatology and Orthopedic, Shanghai Jiao Tong University Affiliated Sixth People’s Hospital, Shanghai 200233, China ^3^Department of Ultrasound in Medicine, Shanghai Jiao Tong University Affiliated Sixth People's Hospital, Shanghai Institute of Ultrasound in Medicine. Shanghai 200233, China
Name and contact information for the trial sponsor {5b}	Contact name: Clinical Research Plan of SHDC Address: 2,Kangding Road, Shanghai, 200041, China Phone code: 021-96886
Role of sponsor {5c}	The sponsor played no part in study design; and will play no part in the collection, management, analysis, and interpretation of data; writing of the report; and the decision to submit the report for publication.

## Introduction

### Background and rationale {6a}

Acupuncture is an ancient medical technique in China with a history of more than 2000 years [1]. Acupuncture therapy involves the insertion of a needle into the body at specific points to prevent or treat various diseases. Although the mechanism of acupuncture remains unclear, its therapeutic benefit has been approved by many western countries and is used as complementary and integrative medicine globally [2–4]. This is exemplified by the fact that Chinese acupuncture was included in the representative list of Intangible Cultural Heritage of Humanity in 2010.There were more than 4000 registered medical practitioners in the Chinese Medicine Board of Australia (CMBA) by 2014 [5]. Acupuncture is also an integral component of conventional therapy in the UK, with approximately 4 million acupuncture treatments having been provided in 2009 alone [6]. In the USA, 6.8% of 22,512 adults reported they will use acupuncture for life [7] and between 2002 and 2012 the number of acupuncture users and licensed acupuncturists increased by 50% and 100% respectively [8].

Traditional Chinese medicine (TCM) merges with naturalistic theories, philosophy, and other alternative disciplines. The common ideology holds that qi is the primary substance and inherent motivation of life. This life force is distributed in the viscera and meridians to promote and stimulate the physiological activities of human body. According to TCM theory, the dysregulation of qi movement is the root cause of diseases. *Deqi is* a complicated sensory composite that accompanies needling stimulation. The onset of deqi is considered a vital phenomenon of acupuncture treatment that determines clinical efficacy. The sensation was first documented in Huang Di’s Canon [9]. Although acupuncture practitioners realize the dominant role of *deqi* in achieving a positive clinical outcome [10–12], and it is taken as a key parameter in many acupuncture studies, current clinical research evidence is insufficient to prove the interaction between *deqi* and clinical value [13–16]. Both wrist-ankle acupuncture (WAA) therapy and Fu’s subcutaneous needling (FSN) therapy are without obvious *deqi* sensation, but still have a curative effect [17, 18].

Today, researchers have not yet reached a consensus on the relationship between *deqi*, arrival of *qi* and needling sensation. Moreover, it remains unclear whether patient’s feelings or practitioner’s perceptions are purely subjective. A large quantity of *deqi* subjectivity research is mainly focus on the scale, effectiveness, and classification of acupuncture sensations [19–22]. Objective studies of *deqi* mainly examine fMRI scans [23, 24], electrophysiology [25, 26], photoplethysmography [27, 28], energy metabolism [29, 30], and biomechanics [31, 32]. However, as mentioned above, these non-invasive techniques have failed to provide a clear understanding of the factors involved in *deqi*. In this study, we will use ultrasound imaging to understand the anatomical structure of the acupoint and its structural and functional integration and to observe the effect of acupuncture at Zusanli (ST36)on antral contraction function under *deqi* and non-*deqi* conditions respectively. This will be done with an ultimate goal to establish the relationship among structure, function, and *deqi* sensations.

### Objectives {7}

Participants who are in acupuncture with the *deqi* sensations group will experience more dramatic changes in the antral contraction frequency (ACF), antral contraction amplitude (ACA), and skin temperature of abdomen than those in the acupuncture group without *deqi* sensations.

The objectives include the following:

1. To determine the effects of ultrasound-guided acupuncture at ST36 on the antral contraction frequency, the antral contraction amplitude, and the skin temperature of the abdomen.
2. To observe and analyze the relationship among the acupoint, the anatomical structure of acupoint area, and the antral contraction function in the  acupuncture group (with *deqi*) and sham acupuncture group (without *deqi*) respectively.
3. To provide standardized and visualized experimental basis for improving the safety and effectiveness of clinical acupuncture techniques.

### Trial design {8}

**Fig. 1:**
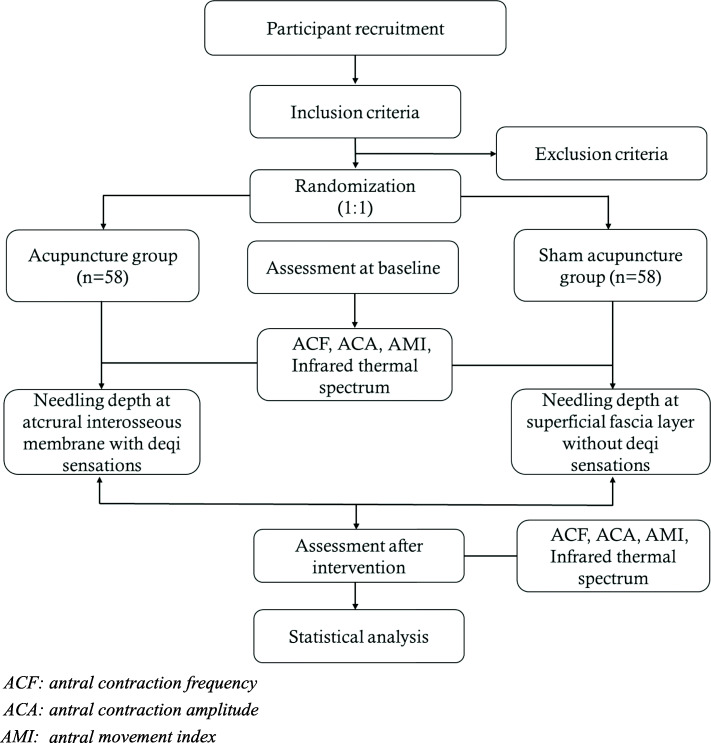
Flowchart of this study

This is a single-site, subjects-blinded, prospective parallel group and randomized controlled trial that conforms to the Standard Protocol Items: Recommendations for Interventional Trials (SPIRIT) 2013 Checklist [33] and standards for Reporting Interventions in Clinical Trial of Acupuncture [34] guidelines for Acupuncture studies. A total of 116 subjects will be included and randomly assigned to the acupuncture group and the sham acupuncture group in a 1:1 allocation radio. The flowchart of the study process is detailed in Fig. 1.

## Methods: participants, interventions, and outcomes

### Study setting {9}

All eligible participants will be accepting the trial in the department of ultrasound in medicine of Shanghai Municipal Hospital of Traditional Chinese Medicine, Shanghai, China.

### Eligibility criteria {10}

Inclusion criteria are as follows: male or female aged 18 to 35 who (1) have a body mass index (BMI) greater than 18.5 and less than 26 and (2) sign the informed consent as a subject voluntarily.

Exclusion criteria are as follows: (1) those who have large or small traumatic injury, skin ulcer, and other skin defects at ST36; (2) participants whose digestive system is found to have an organic lesion after ultrasonic examination; (3) participants with severe primary diseases of cardiovascular, pulmonary, hepatic, renal, and hematopoietic system or severe mental disorder; (4) participants who have been recently taking psychotropic drugs that affect digestion; (5) participants who cannot cooperate with researcher or are unwilling to accept acupuncture intervention; (6) participants who have participated in any other clinical trials in the past 3 months; and (7) participants who are considered inappropriate to be included in this trial by researchers.

### Who will take informed consent? {26a}

All participants will be recruited by advertising in WeChat groups and around universities in Shanghai, China. People who are interested in participating can contact the researcher by calling or adding the WeChat ID, and they will be subsequently evaluated according to the inclusion and exclusion criteria. We guarantee that all participants will be fully informed and understand in detail about the potential benefits and risks of the trial before they sign the informed consent.

### Additional consent provisions for collection and use of participant data and biological specimens {26b}

Not applicable, this trial does not have biological specimens.

## Interventions

### Explanation for the choice of comparators {6b}

In order to comply with the proposed objectives and verify whether depth and intensity of acupuncture will affect the motility of gastric antrum, we take the intervention of shallow acupuncture at ST36 as a way of contrast to distinguish from routine acupuncture. The sensations generated by shallow acupuncture are very slight but is similar to the feeling of true acupuncture. Furthermore, to a great extent, it ensures that the participants cannot distinguish the group distribution in appearance, which is beneficial to blind method implementation.

### Intervention description {11a}

All participants are asked to abstain from eating for at least 2 h before the trial. Participants in both groups will receive acupuncture on the right-sided ST36. A thermal infrared imager detects the thermal infrared spectrum of the gastric area skin and observes the antral contraction by ultrasonography.

#### Acupuncture group

Participants will take the supine position while wearing an eye-patch. The right calf and abdomen will be uncovered sufficiently to expose the lower border of the right patella and the space between the navel and xiphoid process respectively. The infrared imaging device will be used to measure the infrared thermal spectrum for 10 min at a lens distance of 1 m from the subject’s abdomen. Subsequently, sagittal scanning will be performed with an abdominal probe placed slightly to the right of the inferior of border of the xiphoid process. The left lobe of the liver, the lower pancreas, the abdominal aorta, and the superior mesenteric artery will be used as anatomical reference points to locate the gastric antrum. From here, the frequency of gastric antrum contraction will be recorded for 10 min continuously.

After sterilizing the skin, ST36 will be punctured at the insertion point under the guidance of ultrasonography until the needle tip reaches the crural interosseous membrane. A qualified and experienced acupuncturist will perform needle manipulation including lifting, thrusting and rotating to obtain the appropriate needling sensations (*deqi* sensation). Then, the researchers will record the frequency of antral contraction for 10 min with the ultrasonic probes and conduct needle manipulation in the 5th minute. After wiping the ultrasound gel off the skin until it thoroughly dried, the researchers will collect infrared thermal spectrum data from the abdomen.

#### Sham acupuncture group

Participants in the sham acupuncture group will be punctured at ST36 with the needle tip reaching no deeper than the superficial fascia and without any needle manipulations. The remaining procedures and recording details are the same as those of the acupuncture group.

### Criteria for discontinuing or modifying allocated interventions {11b}

The sonographer of this study will perform an ultrasound examination of the stomach before the trial to make sure there are no pathologic changes in the stomach. The trial will be discontinued if one of the following situations occurs: a large amount of gas in the gastrointestinal tract that affected the ultrasonic observation, which can result in collecting and analyzing data difficultly, and participants who have acupuncture-related serious adverse reactions during the experiment or failure to complete the study for any other reasons. We will record the details in the report for the early termination of the study.

### Strategies to improve adherence to interventions {11c}

At the beginning of the trial, participants will be informed of the experiment procedure again. They can report their personal feelings at any time during the study, and we will give corresponding feedback as appropriate to increase the interactivity and then improve adherence to interventions.

### Relevant concomitant care permitted or prohibited during the trial {11d}

Participants will not be allowed to eating or drinking during the entire study. In order to avoid the influence of other factors on body temperature, participants should also be prohibited from leaving the bed for free movement.

### Provisions for post-trial care {30}

At the end of the study, we will provide certain amount of subsidy to each subject. If the adverse events occur during the trial that related to the intervention after the expert committee confirms, the responsible department will provide corresponding economic compensation, psychological comfort, and treatment modalities. Before the trial, the participants will be informed that participation in this study is voluntary, they can withdraw from the study at any time for any reasons, and their benefits will not be affected.

## Outcomes {12}

We will assess the function of gastric antrum contraction by visualizing the antral area, frequency and amplitude during the contraction of gastric antrum and assess this data through the antral motility index [35]. Data collected from the ultrasound will be measured by means of recording and playback.

### Primary outcome

Antral contraction frequency (ACF) is the primary outcome of this study. The ultrasound probe will be placed vertically to simultaneously obtain the image of gastric antrum, superior mesenteric artery, abdominal aorta, and left lobe of the liver. ACF will be recorded for 10 min before and after the continuous needling. Frequency of antral contractions = number of contractions per 10-min period.

### Secondary outcomes

#### Antral contraction amplitude (ACA)

Antral contraction amplitude (ACA) is an average value calculated as follows: amplitude (%) = (antral area at relaxation − antral area at contraction)/antral area at relaxation × 100. The minimum and maximum cross-sectional areas of the antrum will be measured during contractions and relaxations a minimum of 4 times to calculate the amplitude of antral contractions. The antral area will be measured by the Bolondi method [36] which takes the length diameter (*L*) and the width diameter (*W*) of sagittal plane of the antral as parameters for the computational formula (*A* = *L* × *W* × *π*/4).

#### Antral motility index (AMI)

The antral motility index (AMI) is represented as the antral contraction frequency (ACF) multiplied by the antral contraction amplitude (ACA).

#### Infrared thermal spectrum

The infrared thermal spectrum will be measured for 10 min before and after needling by FOTRIC 225 medical infrared imager. We will take the value of the highest temperature within 10 min as the final result of skin temperature in the abdominal region.

#### Ultrasonic anatomical diagram of ST36

A high-frequency ultrasound probe (18 MHz) will be used to conduct spatial positioning at the acupoint before acupuncture. The vertical distance between important structures (anterior tibial artery, deep peroneal nerve and crural interosseous membrane) and the skin will also be measured. The probe will also be used to collect a static and dynamic ultrasonogram of the *deqi* sensations.

#### Clinical evaluation scale for subject sense of acupuncture

This scale is the Chinese version of the Massachusetts General Hospital Acupuncture Sensation Scale (MASS) which has previously been shown to be accurate and reliable [37]. This scale is composed of a main scale and 2 supplementary scales for measuring the spread of the acupuncture sensations and anxiety. At the end of the trial, participants will be asked to fill in the C-MASS form (see additional file 1) to evaluate the intensity of 12 acupuncture sensations on a 0–10-cm VAS, where 0 represents “none” and 10 represents “intolerably strong sensation.” The participants can assess any other sensations by an additional blank row included at the end of the form. The MASS index will then be calculated.

#### Clinical evaluation scale for acupuncturist sense of acupuncture

This scale (see additional file 1) is aimed at the evaluation of the acupuncturist’s feeling of sensations conducted through the needle tip. This data will be collected at the end of the experiment and contains 2 aspects: property and corresponding layer of needling sensations. This scale has reliable content validity and contains 6 sensory items from the experts intentional survey [38].

### Participant timeline {13}

**Fig. 2:**
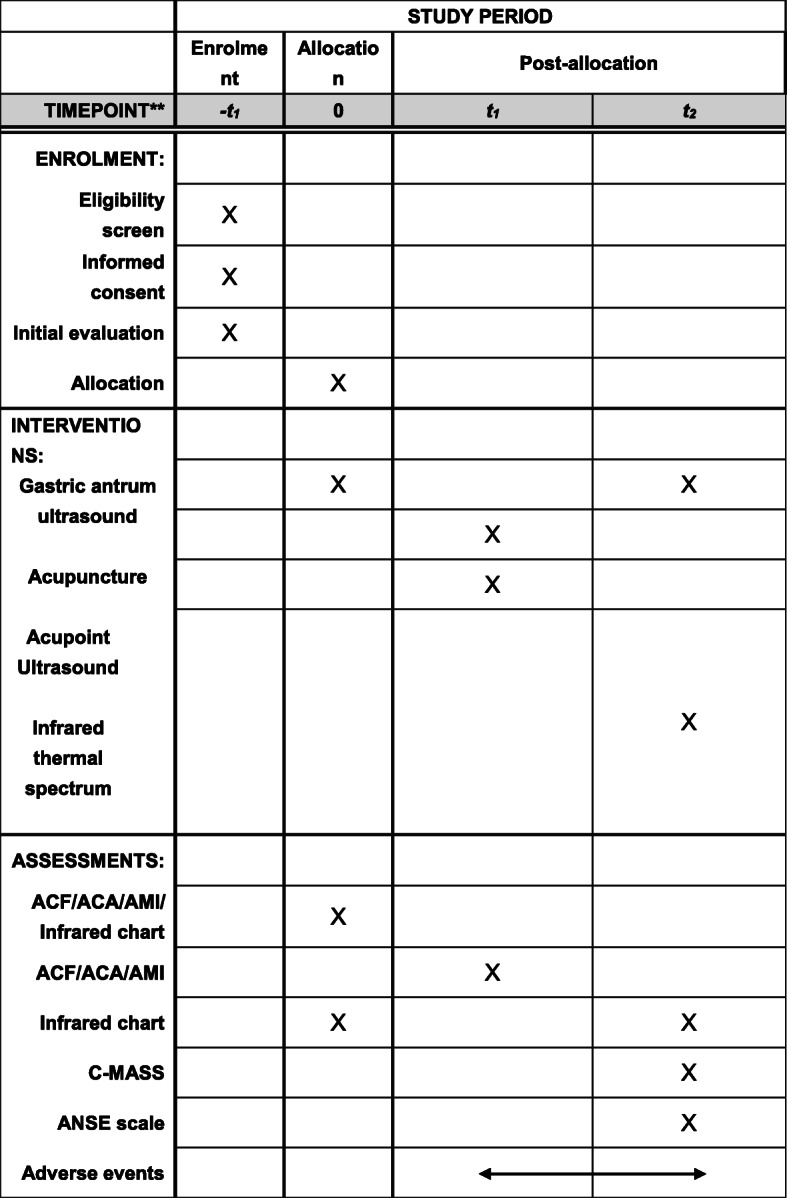
The recommended SPIRIT figure with the participant timeline. 0_=_ baseline_=_ pre-acupuncture; *t*_*1* =_ during acupuncture; *t*_*2*_ = post-acupuncture; ACF, antral contraction frequency; ACA, antral contraction amplitude; AMI, antral movement index; C-MASS, Chinese-Massachusetts General Hospital Acupuncture Sensation Scale; ANSE scale, acupuncturist needling sensations evaluation scale

The timing of intervention and data collection is detailed in Fig. 2.

### Sample size {14}

The sample size was estimated using the Power Analysis and Sample Size (PASS version 15) software. According to our pilot trial, we anticipate that the change in the frequency of antral contraction will be more pronounced in the acupuncture group than in the sham acupuncture group and that we will detect a difference of 30% between effective rates in the 2 groups (70% in the acupuncture group compared with 40% in the sham acupuncture group). On the basis of a 2-sided alpha level of 0.05 and a power level of 90% with a 10% dropout rate, we calculated that our trial will require a sample of 116 participants (58 in each group).

### Recruitment {15}

To achieve adequate participant enrolment to reach target sample size. We will post recruitment advertisements message on WeChat groups of universities in Shanghai and put up the advertisements posters in universities to recruit subjects. Universities are the best strategic places to recruit participants needed for this experiment.

## Assignment of interventions: allocation

### Sequence generation {16a}

This experiment will adopt the method of block randomization. An independent researcher who is not in contact with the participants will use the software SPSS23.0 to generate a random number table to divide the 116 eligible participants into an acupuncture group and sham acupuncture group with a 1:1 ratio.

### Concealment mechanism {16b}

After the sequence number is generated, the researcher will make allocation cards and seal each card in an opaque envelope. The envelope will be kept by the same researcher responsible for sequence generation.

### Implementation {16c}

An independent researcher that is blinded to the study protocol will generate the allocation sequence. According to the timing sequence of the participant registration for the trial, another researcher will arrange the participants into different groups and inform the acupuncturists of the group assignments.

## Assignment of interventions: blinding

### Who will be blinded {17a}

This is a single-blinded (participants-assessor-blinded) trial. Participants will be asked to wear an eye mask during the whole intervention in an enclosed space. All researchers except the acupuncturists and sonographer will be blind to the group assignment, including statisticians, outcome assessors, and data analysts. We will attempt to validate the successful implementation of the blinding method. All participants will be told that the presence or absence of acupuncture sensation is normal. Furthermore, all researchers will accept training on the RCT specification before the trial and will strictly adhere to the separation principle of each department.

### Procedure for unblinding if needed {17b}

Unmasking is not needed. If a participant asks to be unblinded out of curiosity rather than the adverse reactions because of intervention, he or she will be informed of the group allocation after the trial.

## Data collection and management

### Plans for assessment and collection of outcomes {18a}

The measurement and collection of this study’s outcomes include ultrasound observation video of antral motility, ultrasonic image of ST36, and infrared chart of stomach before and after acupuncture. ACF, ACA, and AMI are the three indicators of antral motility, which will be continuous monitoring by ultrasound diagnostic equipment to ensure that there is sufficient playback data for analysis.

#### Plans to promote participant retention and complete follow-up {18b}

Follow-up of subjects is not required in this study. This trial is an observation of the immediate effects of acupuncture intervention without involving any longer follow-up evaluation. We will collect ultrasound and infrared thermal imaging data before and after acupuncture intervention. Although participants who do not receive needling for any reason, their ultrasonic data of gastric antrum will also be collected, because we need to use ultrasound imaging technology to help determine whether the participants meet the inclusion criteria.

### Data management {19}

We will use a clinical trial management platform called ResMan to manage the original data, which will be collected and double-entered by blinded assessor. The data management system will be tested before the officially launched and will be protected by password. The original data of participant will be entered within 1 week after finishing the trial. Data supervisor will check every trial data at least once a month to make sure that they will be recorded accurately, timely, and completely.

### Confidentiality {27}

This protocol, CRFs, and other documents and materials related to the study will be kept strictly confidential and will not be disclosed to third parties except with the consent of the Principal Investigator. Any public report on this study will not disclose the subjects’ personal identity, and we will make every effort to protect the privacy of participants’ personal medical information within the sphere permitted by law. The staff in this study is also bound by this agreement.

### Plans for collection, laboratory evaluation, and storage of biological specimens for genetic or molecular analysis in this trial/future use {33}

Not applicable, this trial does not have biological specimens.

## Statistical methods

### Statistical methods for primary and secondary outcomes {20a}

Data analyses will be performed with the statistical software SPSS23.0 by an independent statistician. The analysis will include an intention-to-treat(ITT) analysis, including data for all participants who dropped out of the trial. Descriptive statistical analysis will be used for all demographic and clinical characteristics of subjects such as sex, age, and weight. We will validate the homogeneity of demographic characteristics and examine the variables between the 2 groups. The mean ± standard deviation will be used for description of the continuous variables. Qualitative data will be expressed in terms of frequency and percentage.  Student's t-test will be used to compare the measurement data between the 2 groups. Rank-sum test is used for ranked data, while the chi-squared test is adopted to analyze categorical data. The significance level used for statistical analysis with 2-tailed testing will be 5%.

### Interim analyses {21b}

Not applicable. Interim analyses will not be performed in this study.

### Methods for additional analyses (e.g., subgroup analyses) {20b}

Not applicable. The trial has no plan for additional analyses.

### Methods in analysis to handle protocol non-adherence and any statistical methods to handle missing data {20c}

All data in this study will be analyzed according to the principle of ITT to reduce deviation, including the subjects who withdraw from the study during the study period. The missing data will be substituted into the subjects’ baseline data for final data analysis.

### Plans to give access to the full protocol, participant level-data, and statistical code {31c}

The data sharing process will comply with the principles of good practice, and the data sharing will be conducted in accordance with the regulatory requirements.

## Oversight and monitoring

### Composition of the coordinating center and trial steering committee {5d}

Not applicable. There is no coordinating center and trial steering committee of this study.

### Composition of the data monitoring committee, its role and reporting structure {21a}

To guarantee the quality of this RCT, it will be carried out in a professional setting at the Shanghai Municipal Hospital of Traditional Chinese Medicine. We will promptly input the data on ResMan website. Working as the data monitoring team, the Clinical Research Center of Drugs of Shanghai University of TCM will identify problems in the trial, examine collected data, and control bias. A qualified clinical trial specialist will be invited to monitor this study.

### Adverse event reporting and harms {22}

Any adverse events related to the intervention will be reported by participants and practitioners. Details of each adverse event will be registered by the Scientific Research Supervision group of the Shanghai Municipal Hospital of Traditional Chinese Medicine and chronicled in a case report form. Adverse events include any symptoms or diseases that are unexpected throughout the duration of the RCT. All subjects in the trial will receive a certain amount of compensation.

### Frequency and plans for auditing trial conduct {23}

The monitoring Committee is entitled to review and inspect the relevant materials of the trial and supervise the implementation process by telephone, email, or video regularly to ensure that the study is carried out according to the protocol.

### Plans for communicating important protocol amendments to relevant parties (e.g., trial participants, ethical committees) {25}

Any modifications that may have an impact on the study, potential benefit to patients, or affect patient safety, including changes of the study objective, study design, patient population, sample size, or study procedure will require a formal revision of the study protocol. This will be decided jointly with the monitoring Committee and the Clinical Research Center of Drugs and approved by the Ethics Committee.

### Dissemination plans {31a}

The publication of the outcomes of this study will provide baseline data and acupoint ultrasound images as well as acupoint ultrasound scanning practices procedure. The outcomes may be presented at conferences, symposiums, teaching classes, etc., if applicable.

## Discussion

*Deqi* refers to the patient’s experience of soreness, numbness, distention, and heaviness and the doctor’s sense of a heavy and tight sensation coming from beneath the acupuncture needle, as described vividly in Biao you fu (Odd to Elucidate Mysteries) written by DOU Han-qing in Yuan Dynasty [39], “*Qi* has not been achieved if the feeling is light, unstable and slow; on the contrary, if the feeling is heavy, sinking and tight, the qi can be achieved.” “*Arrival of qi* is just like ups and downs of the fish after swallowing the bait, non-*arrival of qi* is like the profundity of deep and serene ancient hall.” Obviously, feelings under the needle tip like “fishing bite” indicates “*arrival of qi.*” The essence of acupuncture therapy lies in regulating *qi*. *Arrival of qi* is the precondition for *deqi*; meanwhile, *deqi* is the continuation of *arrival of qi* and the premise of acupuncture manipulation [19].

The main objective of our trial is to design a robust trial to study the relationship between *deqi* sensations, the depth of needling, and the acupuncture effect of a specific point. We will overcome the safety challenges presented when an acupuncturist performs needling by observing ionizing radiation damage, as demonstrated in previous acupuncture clinical trials. In order to achieve this goal, we designed a strictly randomized controlled trial using ultrasonic imaging technology with the ability to observe and record in real time, using high-resolution probes to locate acupoints and capture the static and dynamic state of the target structure.

Assessing the *deqi* sensations is an important part of this trial. General understanding of the term *deqi* is not accurate enough to focus solely on patient’s feelings or acupuncturist’s perception as the criteria of *deqi*. Actually, *deqi* is an umbrella term for a group of phenomena, signifying a state of *qi* and blood changing in the body after being stimulated by acupuncture, moxibustion, or acupoints pressure of meridians [40]. Based on that, we will use both subject’s and acupuncturist’s clinical evaluation scales to the assess *deqi* and needling sensations. Furthermore, we will also use a medical infrared imager to measure skin temperature changes on the abdomen, considering that ST36 is the He point of the stomach meridian of foot-yangming.

The key technical problems to be solved in this experiment are as follows: (1) the measurement of thermal infrared imaging is sensitive to ambient temperatures (2) to observe and guide the whole process of needling and obtain a legible ultrasound image of the needle body and (3) the application of the sham acupuncture method. The human skin surface temperature changes very little in a constant temperature environment. So, we will use a highly sensitive and accurate thermometer to monitor the room temperature in real time, in a closed space without convection gas or other extraneous heat source. It is necessary for the needle body to be perpendicular to the acoustic waves in order for optimal propagation. Only by using this orientation can we effectively guide the whole process of the needling. This means acupuncturists will need to penetrate into the skin at a point away from the routine insertion point. Nonetheless, we can also guarantee that the acupuncture area is located with precision. The acupuncturist and sonologist should be trained several times before the trial begins to ensure an effective implementation of the sham acupuncture technique and to be prepared to give reasonable explanations in case of doubts from sham acupuncture group participants.

There are some limitations in this randomized controlled trial. It is inevitable for acupuncturists to know when they are implementing the real or sham acupuncture treatment and its possible effects. To minimize suggestive bias from this limitation, we will ask all participants to wear an eye-mask. This trial is the primary study into the effect of a specific acupoint in relation to the *deqi* condition. In further studies, we may consider a multicenter clinical trial with patients. We expect that this study will provide more credible evidence for the effect of *deqi* sensations and the specificity of ST36 on gastric antrum contraction function and make up for the deficiency of current clinical trials focused on the methodology of acupuncture.

## Trial status

We started recruiting participants in December 2020 and the recruitment is expected to end late 2021. The protocol version 1.1, which has approved by the Ethics Committee of Shanghai Municipal Hospital of Traditional Chinese Medicine on 4 November 2020.

## Supplementary Information

**Additional file 1.** C-MASS form. Clinical Evaluation Scale

## Data Availability

All data generated or analyzed during this study are included in this published article and its supplementary information files.

## Abbreviations

CMBA: Chinese Medicine Board of Australia
TCM: Traditional Chinese medicine
WAA: Wrist-Ankle acupuncture
FSN: Fu’s subcutaneous needling
ACF: Antral contraction frequency
ACA: Antral contraction amplitude
SPIRIT: Standard Protocol Items Recommendations for Interventional Trials (*newline*) AMI: Antral movement index (*newline*) BMI: Body Mass Index (*newline*)
MASS: Massachusetts General Hospital Acupuncture Sensation Scale
C-MASS: Chinese-Massachusetts General Hospital Acupuncture Sensation Scale
PASS: Power Analysis and Sample Size (newline) ITT: Intention to Treat
