# Supplementary material for: Effect of acupuncture at Zusanli (ST36) point on antral contraction function under ultrasound guidance: study protocol of a randomized controlled trial
Source: Trials. 2021 Nov 15;22:803. doi: 10.1186/s13063-021-05704-9 (PMC8591957; doi:10.1186/s13063-021-05704-9)
Supplement: Supplementary file 1 — Additional file 1. C-MASS form. Clinical Evaluation Scale [file 13063_2021_5704_MOESM1_ESM.pdf]

Clinical Evaluation scale for acupuncturist sense of acupuncture	
Please chose one or more of the following sensations that correspond to your feeling of *deqi* under the needle tip and write down the duration and corresponding layer of the sensation.	
property	anatomical layer and duration
Heavy tight and not fluent	
Light and smooth	
Heaviness under the needle tip	
The needle body is drawn and pulled internally	
Feel loose (as tofu) under the needle tip	
Insentience	

医者针刺得气感评价	
请您在以下感觉中，勾选出一个或多个符合您在针刺时针下得气的感觉，并写出感觉出现持续的时间	
性质	出现的层次
沉紧涩滞	
徐和轻滑	
针下沉重	
针体内吸、牵拉	
针下有舒松感（松散如插豆腐）	
无感觉	

	0	1	2	3	4	5	6	7	8	9	10
**soreness**	**none**		**mild**			**moderate**			**strong**		**unbearable**
**aching**	**none**		**mild**			**moderate**			**strong**		**unbearable**
**deep pressure**	**none**		**mild**			**moderate**			**strong**		**unbearable**
**heaviness**	**none**		**mild**			**moderate**			**strong**		**unbearable**
**fullness/distention**	**none**		**mild**			**moderate**			**strong**		**unbearable**
**tingling**	**none**		**mild**			**moderate**			**strong**		**unbearable**
**numbness**	**none**		**mild**			**moderate**			**strong**		**unbearable**
**sharp pain**	**none**		**mild**			**moderate**			**strong**		**unbearable**
**dull pain**	**none**		**mild**			**moderate**			**strong**		**unbearable**
**warmth**	**none**		**mild**			**moderate**			**strong**		**unbearable**
**cold**	**none**		**mild**			**moderate**			**strong**		**unbearable**
**throbbing**	**none**		**mild**			**moderate**			**strong**		**unbearable**
**other (subject defined)**	**none**		**mild**			**moderate**			**strong**		**unbearable**

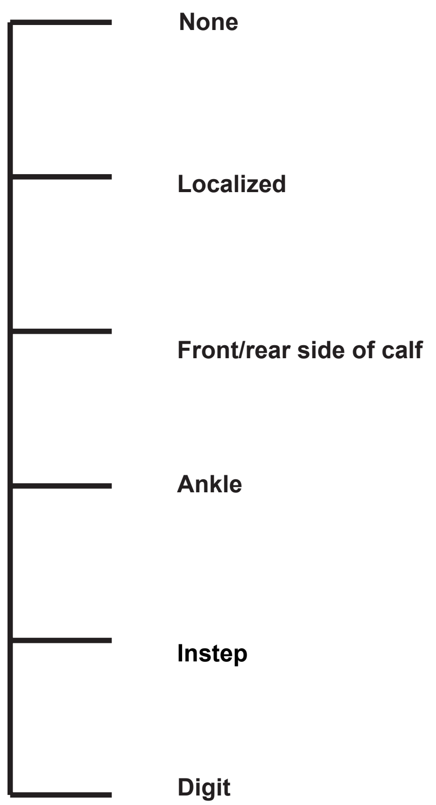

FIG. 2. The supplementary MASS scale entitled "MGH Acupuncture Sensation Spreading Scale." Subjects use this scale to rate the spreading/radiation *de qi* sensations along their applied acupuncture point.

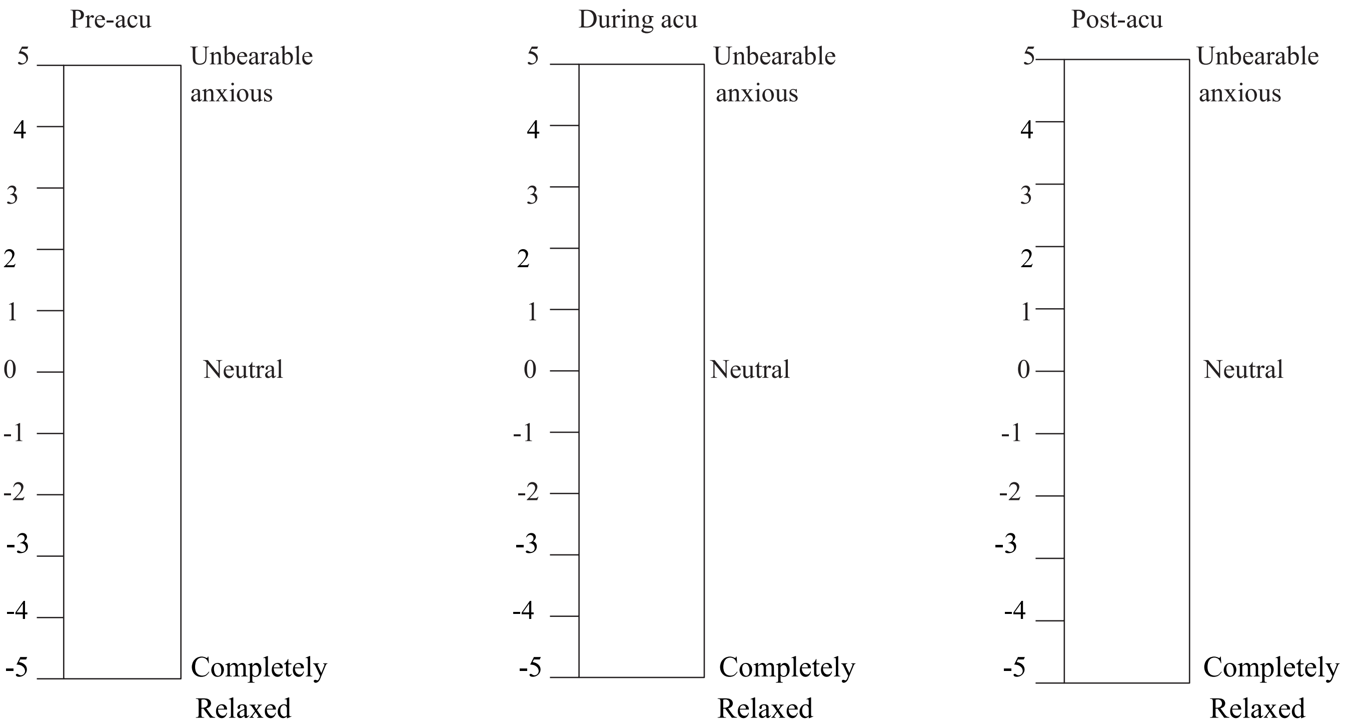

FIG. 3. The second supplementary "Mood Scale," on which subjects rate pre-, during, and post acupuncture treatment anxiety on a 10.0-point continuum (ranging from —5 to 5), with evenly spaced terms "completely relaxed," "neutral," and "unbearably anxious."

受试者针感临床评价量表

填写说明

针刺操作的过程中，通常会因为针灸针的刺入、采用电针以及针灸医师的手法操作激发患者的一些感觉体验。一般将针灸针入皮引发的疼痛称为“刺皮感”；将针灸针在穴位深部引发的感觉称为＂针感＂。这份问卷针对针刺过程中引发的感觉进行记录，可以使我们全面的了解您对针刺的反应。请您根据自己的体会，在记录者的指导下，认真阅读题目问题及填写要求，完成问卷中的问题。谢谢您的配合！

一、在接受针刺的整个过程中，您是否出现以下列出感觉的一种或几种，请根据您自身的体会，在标尺对应的位置上用**“X”表示，0为最低分，10为最高分；若以下选项均无符合您的体会，可在最后“其他”选项进行补充，数目不限。**

0 1 2 3 4 5 6 7 8 9 10

酸痛 |____|____|____|____|____|____|____|____|____|____|

无 轻微 一般 强烈 不能忍受

0 1 2 3 4 5 6 7 8 9 10

疼痛 |____|____|____|____|____|____|____|____|____|____|

无 轻微 一般 强烈 不能忍受

0 1 2 3 4 5 6 7 8 9 10

深部压觉 |____|____|____|____|____|____|____|____|____|____|

无 轻微 一般 强烈 不能忍受

0 1 2 3 4 5 6 7 8 9 10

沉重 |____|____|____|____|____|____|____|____|____|____|

无 轻微 一般 强烈 不能忍受

0 1 2 3 4 5 6 7 8 9 10

胀满 |____|____|____|____|____|____|____|____|____|____|

无 轻微 一般 强烈 不能忍受

0 1 2 3 4 5 6 7 8 9 10

刺痛 |____|____|____|____|____|____|____|____|____|____|

无 轻微 一般 强烈 不能忍受

麻木

0 1 2 3 4 5 6 7 8 9 10

无 轻微 一般 强烈 不能忍受

锐痛

0 1 2 3 4 5 6 7 8 9 10

无 轻微 一般 强烈 不能忍受

钝痛

0 1 2 3 4 5 6 7 8 9 10

无 轻微 一般 强烈 不能忍受

热感

0 1 2 3 4 5 6 7 8 9 10

无 轻微 一般 强烈 不能忍受

冷感

0 1 2 3 4 5 6 7 8 9 10

无 轻微 一般 强烈 不能忍受

跳动感

0 1 2 3 4 5 6 7 8 9 10

无 轻微 一般 强烈 不能忍受

其他

0 1 2 3 4 5 6 7 8 9 10

无 轻微 一般 强烈 不能忍受

二、在接受针刺的整个过程中，您是否出现**针感扩散的现象，请根据您自身的体会，在标尺对应的位置上用“X”表示。**

无

针刺处（局部）

大腿后侧

小腿后侧

脚踝

足趾

附表1 针感扩散量表

三、在接受针刺的整个过程中，请您对**自身的情绪进行打分，5为极度紧张，0为一般，-5为完全放松。请根据您自身的体会，在标尺对应的位置上用“X”表示。**

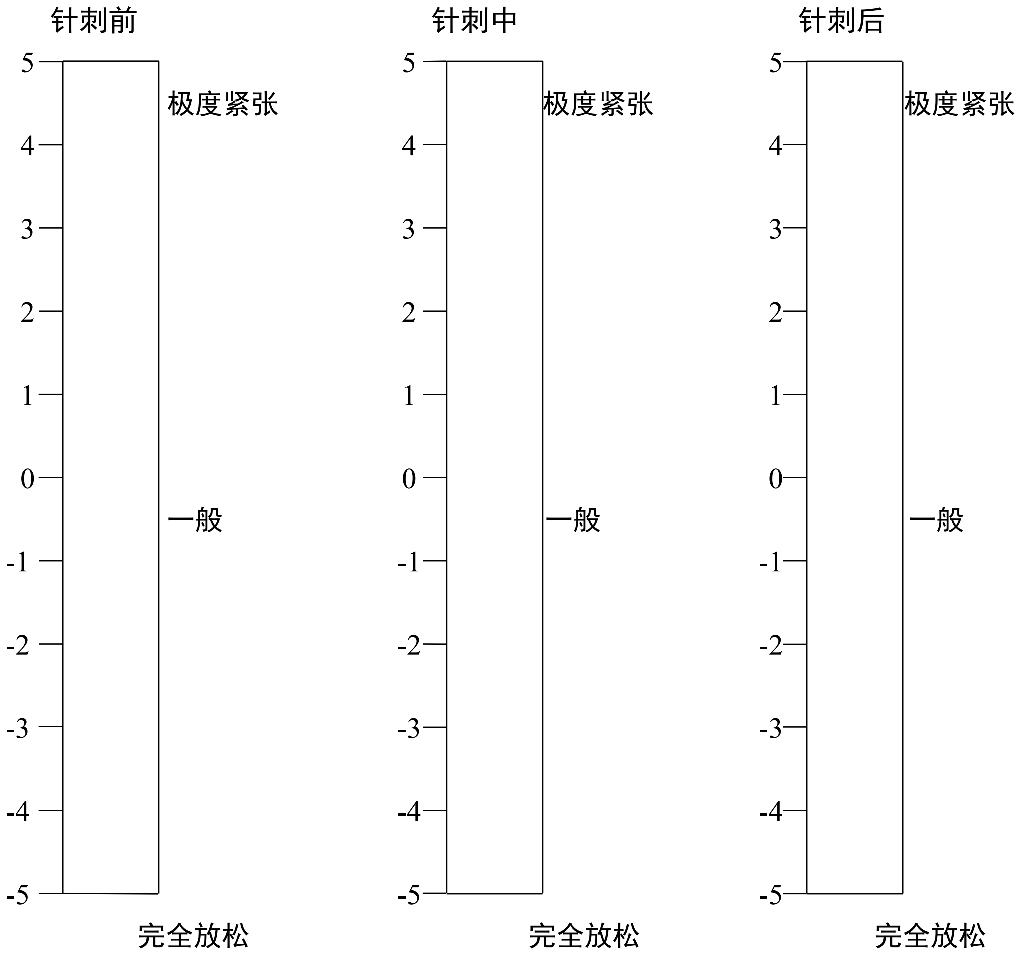

附表2　情绪量表

Informed Consent Form-Information page

Dear participant:

We are inviting you to participate in a randomized controlled trial of effect of acupuncture at Zusanli Point on antral contraction function under ultrasound guidance. Before you decide whether to participate in this study, please read the following contents carefully as much as possible. If you prefer, you can also discuss with your relatives or friends, or ask your doctor for explanation to help make a decision.

Research introduction

1. Research background and purposes

Acupuncture as an important branch of traditional Chinese medicine (TCM) has been used since ancient times because of its remarkable clinical efficacy. With the international development of acupuncture, the standardized requirements are produced. On the basis of current situation of the development of acupuncture standardization, domestic scholars and institutions clarify the theory of acupuncture standardization that facing problems like method is insufficient, lack of generic technology and talent training, evaluation system incomplete. The visualization and standardization of acupuncture is one of the main reasons for the unstable clinical efficacy. Therefore, it is necessary to further study the standardization of acupuncture treatment, improving the accuracy and standardization of acupuncture manipulation. Exploring the standardization of acupuncture technology is the top priority of current research.

Based on the previous work, this research optimized the acupuncture manipulation under ultrasonic visualization and paid attention to the whole process of acupuncture dynamically by ultrasonic. On this basis, a randomized controlled study was conducted to observe the immediate effect of needling *deqi* at Zusanli point on gastric antrum contraction frequency and amplitude, epigastric skin temperature under the guidance of ultrasound, while exploring the relationship between them. To provide standardized and visual experimental evidence for improving the safety and effectiveness of clinical acupuncture technology.

A total of 120 participants are expected to participate voluntarily. This research was reviewed by the Ethics Committee of Shanghai Municipal Hospital of Traditional Chinese Medicine, complying with the relevant national laws and regulations and the Helsinki Declaration and other ethical principles to protect the rights of subjects.

2. Who are not suitable for the trial?

(1) those who have large or small traumatic injury, skin ulcer and other skin defects at

Zusanli point;
(2) participants whose digestive system is found to have an organic lesion after ultrasonic examination;
(3) participants with severe primary diseases of cardiovascular, pulmonary, hepatic, renal and hematopoietic system or severe mental disorder;
(4) participants who have been recently taking psychotropic drugs that affect digestion;
(5) participants who cannot cooperate with researcher or are unwilling to accept acupuncture intervention;
(6) participants who have participated in any other clinical trials in the past 3 months;
(7) participants who are considered inappropriate to be included in this trial by researchers.

3. What will be needed if participate in the research?

3.1 Before enrolling in the study, you will receive some assessments to determine whether you can participate in the study. The doctor will ask and record your medical history and conduct an ultrasonic examination for you.

3.2 If you meet the inclusion criteria， the investigator will arrange for acupuncture or placebo acupuncture according to the randomization. Patients who participated in the study had a 50% probability of being assigned to one group. Both two groups used the same acupuncture manipulation to make needle point arrived at different depth and the anatomical level. You will be asked to abstain from eating for at least 2 hours before the trial. During the needling manipulation, the doctor will ask you about the needle sensations and give a record so please timely and objectively reflect your feelings toward the doctor. The intervention will be lasted for 20min every time.

3.3 Other matters that you need to cooperate with: You should follow the doctor's instructions, including receiving acupuncture intervention and relevant examinations as required, cooperating with medical staff to record, etc. 24 hours before receiving acupuncture intervention, you should not take drugs that affecting neural activity, blood vessels and digestion. Do not shower, drink alcohol or smoke 2 hours before acupuncture. Do not wear tights and apply cosmetics to exposed areas. If you have any other medications that you have to take, please let your doctor know. If you do need other treatments during the study, please contact your doctor. Keep your mood comfortable during treatment.

4. Benefit from participating in the study

You may benefit from the research. The benefits include the knowledge you have

about your own health and the potential for better treatments to benefit patients with related diseases. You will get good medical care during the study.

5.Risk of participating in the study

If there are any discomfort during the study, or a new change in the condition, or any unforeseen conditions, you should notify your doctor promptly and he will make corresponding judgments and medical treatments. We will do our utmost to prevent harm which may be caused by the research. If adverse events occur during the clinical trial, the Medical Expert Committee will authenticate whether it is related to the treatment. Corresponding economic compensation will be provided if adverse events are related to the trial.

6. Detailed expense

We will provide you with a certain amount of subject compensation for participating in this study.

7. Is personal information confidential?

Your medical records (research medical records/CRF, physical and chemical examination reports, etc.) will be kept in the hospital. Researchers, sponsor representatives, and ethics committees will be allowed to access your medical records. Any public report about this study will not disclose your personal status. We will make every effort to protect the privacy of your personal medical information to the extent permitted by law.

8. How can I get more information?

You can ask any questions about this research at any time.

Your doctor will leave you his/her phone number to answer your questions.

Your doctor will notify you immediately if there is any important new information during the study that may affect your willingness to continue your research.

9. You can choose to participate or drop out of research voluntarily

Participate in this study is entirely up to you. You can decline to participate the study or withdraw from the study at any time. Your benefits will not be affected and you will not be discriminated or retaliated if you choose to quit.

Your doctor or researcher may discontinue the study at any time for your best interest.

If you withdraw from the study for any reason, you may be consulted about the interventions. You may also be asked to perform laboratory and physical examinations if necessary.

10. What should I do now?

It is up to you to decide whether to participate in this study. You can discuss it with your family or friends before making a decision.

Before you make a decision, please ask your doctor as much as possible until you fully understand.

Thank you for reading the above materials. If you decide to participate in this study, please tell your doctor that he/she will arrange all the research for you.

Please keep this form.

Informed consent form • signature page

Project Name: Effect of Acupuncture at Zusanli Point on Antral Contraction Function under Ultrasound Guidance: A Study for a Randomized Controlled Trial

Version number: V1.1

Version date: September 22, 2020

Agree with the statement

I have read the above introduction to this study and have the opportunity to discuss and ask questions with the doctor about this study.

All the questions I raised were answered satisfactorily.

I understand the risks and benefits that may arise from participating in this study. I know that participating in the study is voluntary. I confirm that I have enough time to consider it and understand that:

· I can ask the doctor for more information at any time.

· I can withdraw from the study at any time without discrimination or retaliation, and medical treatment and benefits will not be affected.

I also know that if I withdraw from the study, I will tell the doctor about the change of the condition and complete the physical examination and physical and chemical examination. This will be very beneficial to me and the whole study.

If I take any other medications for illness, I will ask the doctor for advice beforehand or tell the doctor truthfully afterwards.

I agree with the responsible doctor of the project, the ethics committee of Shanghai Municipal Hospital of Traditional Chinese Medicine, and other departments to check my research data.

I will receive a copy of the signed and dated informed consent form.

Finally, I decided to participate in the study and follow my doctor's advice.

Patient signature：____________________________ Date：__________

Patient contact number：______________________

I confirm that I have explained the details of the study, including the rights, potential benefits and risks, and gave him or her a copy of the signed informed consent.

Researcher signature：_________________________ Date：__________

Researcher contact number：____________________

知情同意书•知情告知页

尊敬的志愿者：

我们将邀请您参加一项“超声引导下针刺足三里穴对胃窦收缩功能的影响”的随机对照研究。

在您决定是否参加这项研究之前，请尽可能仔细阅读以下内容，它可以帮助您了解该项研究以及为何要进行这项研究，研究的程序和期限，参加本研究后可能给您带来的益处、风险和不适。如果您愿意，您也可以和您的亲属、朋友一起讨论，或者请您的医生给予解释，帮助您做出决定。

研究介绍

一、研究背景和研究目的

针灸学作为祖国传统医学的重要分支从古沿用至今，重要原因之一是其临床疗效显著。在针灸面向国际发展前提下，便产生了标准化的需求，国内先后有学者及机构开展针灸标准化的研究，而在近十年针灸标准化发展现状的基础上，厘清了针灸标准化面临的理论方法不足、共性技术缺乏、评价体系不完备、人才培养缺失等情况。但对于针灸的可视化及标准化治疗仍缺乏有力证据，而针灸的可视化和标准化是临床疗效不稳定的主要原因之一。因此，进一步研究针灸治疗的可视化及标准化，提高临床上针灸治疗的准确性及规范性，探索针灸技术的标准化是当下研究的重中之重。

本课题在前期工作基础上优化超声可视化下针刺，采用超声动态关注针刺操作全过程。在客观条件上确保针刺特定穴“得气”，得到客观参数（镜下解剖结构、声像特点、针尖位置，针体通过脂肪层深度、针尖距皮肤的深度、针尖距深部骨骼的距离等），形成针刺得气的客观标准。在此基础上通过随机对照研究观察针刺得气标准化情况下对胃窦收缩的即刻效应，探索超声引导下针刺足三里穴得气对胃窦收缩频率、胃窦收缩幅度和胃脘部皮肤温度的影响，并探讨其间的联系，分别观察针刺足三里穴得气（观察组）与不得气（对照组）的情况下，针尖与穴位区域解剖结构及胃窦收缩功能之间的关系，为提高临床针刺技术的安全性和有效性提供标准化、可视化的实验依据。

本研究将在上海市中医医院进行，预计共有 120 名受试者自愿参加。

本研究项目由上海市中医医院伦理委员会审议，是遵从中国国家相关法规和赫尔辛基宣言等保护受试者权益的伦理原则的。

二、哪些人不宜参加研究？

（1）凡腧穴部位皮肤有感染者，畏针者

（2）近期有胃肠病史或经检查发现有器质性病变者，或影响胃排空疾病者

（3）有心血管、肺、肝、肾和造血系统等严重原发性疾病患者，精神性疾病者
（4）近期服用精神类或影响消化功能药物者
（5）不能配合或不愿意接受针刺者
（6） 3个月内参加过或正在参加其他临床研究者
（7） 研究者认为不宜入选本临床试验者

三、如果参加研究将需要做什么？

1. 在您入选研究前，您将接受医生的询问，以记录您的病史，并为您进行超声检查以确定您是否可以参加研究。

2.若您以上检查符合入选条件，研究者将根据随机抽样结果，安排您接受针刺组或对照组的针刺干预，参加这项研究的患者有50%的概率被分入这两组。针刺组与对照组采用同种针刺方法使针尖到达不同深度和解剖层次，两组均在进食后2小时进行干预，针刺观察期间医生将询问您针刺的感受并给予记录，并请您在针刺期间及时、客观地向医生反映自己的感受，按时填写各项评分表进行评估。

3.需要您配合的其他事项：您应该遵照研究方案进行，包括按要求接受针刺干预、相关检查，配合医务人员记录等，接受针刺干预前24小时不服用影响神经、血管及消化的药物；接受针刺干预前2小时不淋浴、饮酒、吸烟；接受针刺干预时不穿紧身衣裤，暴露部位不涂抹化妆品。如您有其他疾病须继续服用的药物，请您务必告知您的医生。在研究期间如您确实需要其它治疗，请事先与您的医生取得联系。治疗期间保持心情舒畅，尽可能情绪平稳。

四、参加研究的受益

您和社会将可能从本项研究中受益。此种收益包括了您对自身健康状况的了解，以及本项研究可能会产生出更佳的治疗方法以有利于相关疾病的病人。您会在研究中得到良好的医疗服务。

五、参加研究的风险

如果在研究期间您出现任何不适，或任何意外情况，不管是否与针刺干预有关，均应及时通知您的医生，我们将对此作出判断和医疗处理。针灸临床试验中可能出现晕针、滞针等不良事件。若发生晕针反应时立即出针，我们让患者平卧，头部稍低，给以温开水或糖水，一般静卧片刻即能恢复。发生滞针时用手指在滞针部位轻轻叩打，使紧张的皮肤和肌肉缓解，或在滞针的针柄上施灸，或在滞针附近的穴位另刺一针，即可缓解滞针现象。如因单向捻动幅度过大，可将针向相反方向捻转，待针体松动后即可出针。

课题负责单位将尽全力预防和治疗由于本研究可能带来的伤害。如果在临床试验中出现不良事件，医学专家委员会将会鉴定其是否与本治疗有关。经专家委员会认定，不良事件与本试验中的治疗有关，课题组将对于试验相关的损害提供

相应的经济赔偿。

您在研究期间需要按时到我们门诊进行治疗和随访，并做一些理化检查，这些都可能给您造成麻烦或带来不方便。

六、参加研究的补偿

研究单位将提供您参加本项研究一定数额的受试者补偿费。

七、个人信息是保密的吗？

您的医疗记录（研究病历/CRF、理化检查报告等）将完整地保存在医院。研究者、申办者代表、伦理委员会将被允许查阅您的医疗记录。任何有关本项研究的公开报告将不会披露您的个人身份。我们将在法律允许的范围内，尽一切努力保护您个人医疗资料的隐私。

八、怎样获得更多的信息？

您可以在任何时间提出有关本项研究的任何问题。

您的医生将给您留下他/她的电话号码以便能回答您的问题。

如果在研究过程中有任何重要的新信息，可能影响您继续参加研究的意愿时，您的医生将会及时通知您。

九、可以自愿选择参加研究和中途退出研究

是否参加本研究完全取决于您的自愿。您可以拒绝参加此项研究，或在研究过程中的任何时间退出本研究。如果您选择退出此研究，您的受益将不会受到影响，也不会因此而受到歧视或报复。

您的医生或研究者出于对您的最大利益考虑，可能会随时中止您参加本项研究。

如果您选择参加本项研究，我们希望您能够坚持完成全部研究过程。

十、现在该做什么？

是否参加本项研究由您自己决定。您可以和您的家人或者朋友讨论后再做出决定。

在您做出参加研究的决定前，请尽可能向您的医生询问有关问题，直至您对本项研究完全理解。

感谢您阅读以上材料。如果您决定参加本项研究，请告诉您的医生，他/她会为您安排一切有关研究的事务。

请您保留这份资料。

知情同意书•同意签字页

项目名称：超声引导下针刺足三里穴对胃窦收缩功能的影响

版本号：V1.1

版本日期：2020 年 09 月 22 日

同意声明

我已经阅读了上述有关本研究的介绍，而且有机会就此项研究与医生讨论并提出问题。我提出的所有问题都得到了满意的答复。

我知道参加本研究可能产生的风险和受益。我知晓参加研究是自愿的，我确认已有充足时间进行考虑，而且明白：

- 我可以随时向医生咨询更多的信息。
- 我可以随时退出本研究，而不会受到歧视或报复，医疗待遇与权益不会受到影响。

我同样清楚，如果我中途退出研究，我若将病情变化告诉医生，完成相应的体格检查和理化检查，这将对我本人和整个研究十分有利。

如果因患病的需要采取任何其他的药物治疗，我会在事先征求医生的意见，或在事后如实告诉医生。

我同意该项目的负责医生、上海市中医医院伦理委员会等部门代表查阅我的研究资料。

我将获得一份经过签名并注明日期的知情同意书副本。

最后，我决定同意参加本项研究，并保证尽量遵从医嘱。

受试者签名：____________________　　　　日期：______ 年 _____ 月_____日

受试者联系电话：__________________________

我确认已向受试者解释了本研究的详细情况，包括其权利以及可能的受益和风险，并给其一份签署过的知情同意书副本。

研究者签名：__________________　　　　日期：______ 年______ 月_____日

研究者联系电话：_______________________
